# Green and Durable Lightweight Aggregate Concrete: The Role of Waste and Recycled Materials

**DOI:** 10.3390/ma13133041

**Published:** 2020-07-07

**Authors:** Jiyu Wang, Kai Zheng, Na Cui, Xin Cheng, Kai Ren, Pengkun Hou, Lichao Feng, Zonghui Zhou, Ning Xie

**Affiliations:** 1 Shandong Provincial Key Laboratory of Preparation and Measurement of Building Materials, School of Materials Science and Engineering, University of Jinan, Jinan 250022, Shandong, China; 20172110138@mail.ujn.edu.cn (J.W.); 201821200850@mail.ujn.edu.cn (K.Z.); chengxin@ujn.edu.cn (X.C.); renkai.jou@gmail.com (K.R.); mse_houpk@ujn.edu.cn (P.H.); mse_zhouzh@ujn.edu.cn (Z.Z.); 2 School of Civil Engineering and Architecture, University of Jinan, Jinan 250022, Shandong, China,; 3 Jiangsu Marine Resources Development Research Institute and School of Mechanical Engineering, Jiangsu Ocean University, Lianyungang 222005, Jiangsu, China; lichaofeng@jou.edu.cn

**Keywords:** lightweight aggregate concrete, waste, recycled materials, durability

## Abstract

Lightweight aggregate concrete manufactured by solid waste or recycled by-products is a burgeoning topic in construction and building materials. It has significant merits in mitigating the negative impact on the environment during the manufacturing of Portland cement and reduces the consumption of natural resources. In this review article, the agricultural and industrial wastes and by-products, which were used as cementitious materials and artificial lightweight aggregate concrete, are summarized. Besides, the mechanical properties, durability, and a few advanced microstructure characterization methods were reviewed as well. This review also provides a look to the future research trends that may help address the challenges or further enhance the environmental benefits of lightweight aggregate concrete manufactured with solid waste and recycled by-products.

## 1. Introduction

Light weight aggregate concrete (LWAC) has been widely applied in constructions with the merits of lightweight, heat preservation, fire resistance, low shrinkage, and creep resistance under normal conditions [1]. The most essential benefit of LWAC is structural weight reduction. As a result, LWAC is suitable for high-rise and long-span buildings [2]. The density of the LWAC is determined by multiple factors, including aggregate types and grading, water content, mixture design, cement types and contents, w/b ratio, chemical additives, compaction approaches, and curing conditions. In general, the LWAC can be divided into three categories according to the density, namely the low density, medium density, and high density corresponding to the range of 400 to 800 kg/m^3^, 800–1350 kg/m^3^, and 1350–1850 kg/m^3^, respectively. The low density LWAC can be used as non-structural applications, while the high density LWAC can be used as structural applications. The mediate density LWAC can be used in both structural and non-structural applications depending on the field requirements [3].

It was reported that [4] the LWAC was mainly made by artificial lightweight aggregates such as expanded clay, slate, shale, and blast furnace slag. As an environmentally friendly material in the construction industry, the ideal lightweight aggregate should be a sintered core with a nearly spherical shape (with the diameter in a range of 4–14 mm) and a rough surface which is impervious to water, as well as strong features such as low porosity and water absorption. These are beneficial to enhance the bonding between the cement matrix and the aggregate [5] when preparing LWAC. As the stiffness of lightweight aggregate is generally lower than that of the ordinary coarse aggregate, the micro-crack of the lightweight aggregate concrete is smaller than the micro-crack of the normal weight concrete (NWC) [6]. Meanwhile, compared with the NWC, the relatively uniform stress distribution of LWAC at the microscopic level improves its durability in aggressive environments [4,7].

The United States is one of the earliest countries that applied the LWAC. Since 1913, the shale concrete has been successfully prepared and implemented in building bridges (Lightweight-Aggregate-History). In Asia, Japan began to produce and use LWAC in 1955 and then it has been widely used for building urban roads, bridges, railways, and marine constructions [8]. China began to study LWAC from the early 1950s, and the use of ceramisite concrete was the primary research trend. In 1958, the pilot slab of the LWAC was prefabricated in Beijing. Two years later, the first LWAC bridge was built in Pingdingshan, Henan Province. Although the structural LWAC has been widely applied in constructions from the 1970s to the 1980s, the maximum strengths were about 20–30 Mpa, which confined its wide applications in the fields. As a result, how to develop high strength lightweight aggregate concrete has become one of the main tasks of the LWAC development [9].

It is no doubt that the production of high-strength LWAC has significant benefits in modern constructions. However, for the production of high-strength LWAC, the high-quality Portland cement, and artificial lightweight aggregates are the key factors that govern the final properties of LWAC. As well-known, the production of high-quality Portland cement is an energy-intensive process, and the manufacture of LWA also requires a large amount of energy consumption [4], which poses a significant carbon footprint to the environment. Therefore, how to manufacture green high-performance lightweight aggregate concrete has become one of the most important trends for the designing and manufacturing of LWAC.

The primary purpose of developing green high-performance light-weight aggregate concrete is to reduce the carbon footprint. It was reported that the LWAC can be prepared by using recycled materials as aggregate [10] and cementitious binders [11]. As a result, the following two aspects can be considered as the future development trends for the production of green high-performance light-weight aggregate concrete. First, completely or partially replacing Portland cement by industrial waste or recycling materials as the cementitious binders; second, replacing the natural coarse and fine light-weight aggregates by using artificial light-weight aggregate prepared with recycling materials.

## 2. Green Cementitious Binders

Portland cement, which provides good mechanical properties, high economic value, and excellent durability, has been widely used as the cementitious binder in modern constructions [12]. However, the production of Portland cement consumes significant energy, which accounts for 3% of global energy usage [13]. Furthermore, approximately 0.9 tons of carbon dioxide will be released per ton of Portland cement production, which accounts for 5% of anthropogenic CO_2_ emissions [13]. According to the requirements of green production, using low carbon cementitious materials [14] and other supplement cementitious materials [15] are the main approaches in sustainable concrete constructions.

### 2.1. Special Type Cementitious Binders

In order to reduce the energy consumption and improve the properties, special type cement can be used to partially or entirely replace Portland cement in light-weight aggregate concrete, especially for the aggressive environment, such as marine area, and sulfate or chloride attack. With exposure to these environments, calcium aluminate (CA) cement, calcium sulfoaluminate (CSA) cement, and supersulfate (SS) cement have been applied to manufacture durable LWAC with exposure to aggressive environment.

CA has significant merits compared to Portland cement, including the rapid hardening, high temperature and temperature change resistance, chemical attack resistance, biogenic corrosion resistance, impact resistance, and abrasion resistance. Unlike the Portland cement, in which the main oxides are CaO and SiO_2_ with forms of tricalcium silicate (C_3_S) and dicalcium silicate (C_2_S), the main oxides of CAC are CaO and Al_2_O_3_ in the form of monocalciumaluminate (CA) instead of C_2_S and C_3_S. Although the CA cement has an outstanding performance, the high price (four to five times higher than the Portland cement) has significantly confined its wide applications, except some extreme cases, such as high temperature or acid attacking. It was claimed that the high-temperature resistance of CA cement lightweight aggregate concrete is better than the normal Portland concrete. This study compares the high-temperature resistance of CA/expanded clay aggregate LWAC and OPC/natural aggregate concrete. The results showed that after exposure to 1000 °C, the residual mechanical properties of the CA/expanded clay aggregate LWAC is better than the OPC/natural aggregate concrete [16].

Apart from CA cement, calcium sulfoaluminate (CSA) cement generally has a higher strength in the early and late stages compared to OPC [17,18]. CSA-based LWAC has good freeze-thaw and chemical attacking resistance with exposure to seawater, sulfate, chloride, magnesium, and ammonium salts [19]. CSA cement is mainly composed of ye’elimite ($C_{4}A_{3}\hat{S}$), belite ($C_{2}S$), calcium sulfate ($C\hat{S}$), and aluminoferrite ($C_{4}AF$) [20]. The ye’elimite will react with water to form ettringite ($C_{6}A{\hat{S}}_{3}H_{32}$) and aluminum hydroxide ($AH_{3}$), shown as the following equations [20], which contribute to the early-age strength development [21,22,23,24]:

(1) $$C_{4}A_{3}\hat{S}+2C\hat{S}H_{2}+34H\to C_{6}A{\hat{S}}_{3}H_{32}+2AH_{3}$$

(2) $$C_{4}A_{3}\hat{S}+6CH+8C\hat{S}H_{2}+74H\to 3C_{6}A{\hat{S}}_{3}H_{32}$$

**Figure 1:**
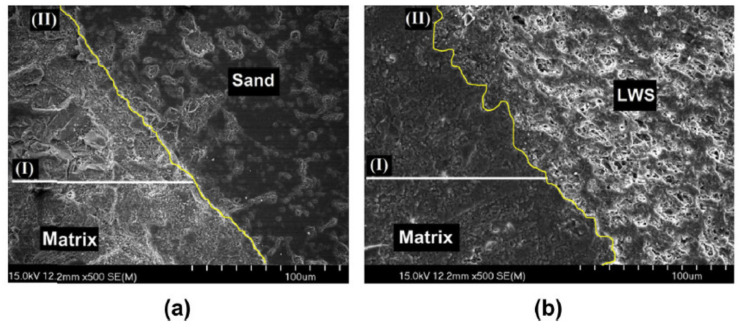
SEM images at 91 days for two different OPC-CSA mortar mixtures: (**a**) interfacial transition zones (ITZ) surrounding sand for OPC-CSA mixture made without light-weight sand (LWS) and (**b**) ITZ surrounding LWS for OPC-CSA mixture containing 20% LWS replacement [19], with copyright permission from Elsevier.

The excellent corrosion resistance of CSA results from the formation of a dense pore structure according to these reactions. A previous study has evaluated the interactions between the OPC, CSA in LWAC [19]. In this study, all samples were prepared with a fixed water/binder ratio of 0.4 and a sand-to-binder ratio of 2.6. The SEM images, shown in Figure 1, compared the interfacial transition zones (ITZ) of the normal sand and light-weight sand (LWS). The moisture curing was not used to enlarge the effect of the LWS on the ITZ. Compared to the OPC-CSA mixture with normal sand, the paste matrix (Figure 1a) around the OPC-CSA mixture LWS containing 20% LWS is more uniform and has smaller pores (Figure 1b). For the LWS sample, the interfacial boundary between the LWS and the paste matrix was not that distinctive compared to the normal sand. This suggests a better interfacial bonding between the paste and the aggregate and leads to the increase of the compressive strength of the LWS-containing OPC-CSA mixture [25].

Because of the low heat of hydration and the excellent corrosion resistance with exposure to aggressive environments, supersulfate cement intrigues broad interests in recent years. Mixed with blast furnace slag, calcium sulfate, and alkaline activators, supersulfate cement showed its great potential as a promising binder to prepare durable concrete with exposure to the aggressive environment [12]. The effects of the blast furnace slag containing various alkali activators on the hydration and strength evolution of supersulfate cement were investigated [26]. It was stated that the chemical composition of the slags has a significant effect on the hydration process, and thus influence the volume stability of the concrete. It was found that, by mixing aluminum sulfate and calcium hydroxide with the furnace slag, the amount of the ettringite has increased, which increases the 28 days strength of the supersulfate cement, although it had little contribution to the early age compressive strength. A similar phenomenon has been confirmed by Masoudi [27].

One of the most impressive merits of the supersulfate cement LWAC is its environmental resistance with exposure to chloride or sulfate attacking. Grounds investigated the resistance of decomposed ettringite to sulfate solutions when supersulfate cement samples are exposed to sulfate [28]. The prepared samples were stored at 25 °C for 28 days and 6 months, respectively. After soaking in distilled water, deionized water, and several sulfate solutions for 6 months, the core and surface areas of the LWAC were analyzed by DTG and XRD. The results showed that the supersulfate cement is resistant to sodium sulfate and calcium sulfate solutions but decomposed in a magnesium sulfate solution. This environmental resistance is related to the lack of calcium hydroxide and the combination of a large amount of free alumina and ettringite during the hydration process. Cerulli used other methods and also proved that the supersulfate cement exhibits excellent environmental resistance to sulfate attack [29].

Although the CA, CSA, and SS cement are considered to have application prospects because of its low carbon dioxide emissions and low energy requirements, it is worth noting that the challenges from academic and practical perspectives are mainly the high manufacturing cost and the lack of understanding the phase formation and hydration process of the mineral contents, and the unpredictable long-term durability.

### 2.2. Recycling Materials as Cementitious Binders

It has been widely recognized that Portland cement production not only consumes tremendous energy but also contributes to significant greenhouse gas emissions. As a result, the search for sustainable and low-carbon cementitious materials to replace Portland cement is burgeoning in recent years. The use of supplementary cementitious materials (SCMs) in normal concrete constructions has been investigated for several decades and showed a few promising benefits to enhance the mechanical properties and durability of concrete materials. However, the effects of the SCMs on the properties and durability of LWAC have yet been systematically investigated, especially by using the solid recycling wastes as SCMs or low-carbon cementitious materials.

In order to study the effect of different SCMs on the carbonization resistance of LWAC, Bogas [30] selected silica fume (SF), fly ash (FA), and lime powder in a lab experiment to compare the results through accelerated carbonization process. In this study, four types of lightweight aggregates were used to prepare LWAC samples, two of which were from Portuguese expanded clay aggregates, one from the United Kingdom sintered fly ash aggregate, and one from the United States expanded slate aggregate. In this study, the LWAs were immersed in water for 24 h beforehand, and various types and contents of SCMs were selected and mixed with lightweight aggregates according to different mixture proportions. The results showed that the SF contributes little to the refinement of concrete pores and may have poor dispersion. The reduction of the carbonize materials and the light densification of the matrix resulted in the increasing carbonization coefficient with increase SF content. Compared with FA concrete, the compressive strength of lime powder concrete was slightly lower, because there was the little pozzolanic reaction in the lime powder concrete. On the other hand, even if the lime powder did not contribute to the CH consumption [15], the amount of the carbonize substances is reduced because of the substitution of lime powder for cement. In order to better understand the possible effects of the mineral admixtures on the carbonization resistance of concrete, it is necessary to analyze not only their role as a substitute material but also their role as supplementary materials.

Ground granulated blast slag (GGBS) as SCMs can partially replace cement, which can improve the workability of concrete by 30% compared with OPC concrete [31]. It is more advantageous to apply it to lightweight aggregate concrete because the specific gravity of GGBS is relatively lower than the Portland cement, suggesting that the replacement of cement by the GGBS (mass) can further reduce the density of concrete. Shafigh et al. [31] and Mo et al. [32] investigated the high volume slag as a replacement of cement to manufacture LWAC by using oil palm shell as coarse aggregate and GGBS as auxiliary cementing material instead of Portland cement to produce green lightweight structural concrete under different conditions. They found that, although with the replacement of Portland cement by 70% had resulted in a reduction of the compressive strength, the properties of the LWAC still meet the structural requirement. However, as the age increased, the compressive strength decreased. Meanwhile, cement consumption and CO_2_ emissions can also be significantly reduced by this substitution. It is worth mentioning that the slag had no obvious effect on the bond strength of concrete of the LWAC [33].

In addition to these conventional SCMs, a few other cementitious materials use for LWAC comes into our views. Lynn [34] was concentrated on the potential of sewage sludge ash (SSA) in the concrete. As a by-product of water treatment, SSA can be used as a cementing component together with a Portland cement clinker and FA to achieve a proper gradation size distribution on the ground.

Bui et al. [1] studied the waste paper sludge (PS), one kind of recycled material, which is a replacement for the cement to enhance LWAC’s quality. FA-based geopolymer (GP) concretes were produced by using a mixture of basaltic pumice (BP) aggregates and fly ash (Class F) for lightweight concrete production, which has several important advantages such as low energy necessity, especially in term of earthquakes [35].

The application of SCMs to ordinary concrete has obtained relatively good application rules and application values. However, the use of these reused SCMs for lightweight aggregate concrete and special concrete under extreme environments is still in the experimental stage. The practical ways and effects need long exploration, especially insufficient research on its durability. An extensive systematic analysis on SSA in concrete and concrete-related products showed that this material had the potential for some different forms, such as lightweight aggregate production and fine aggregate [36,37,38,39,40,41].

## 3. Green Aggregates

Reusing waste materials from agricultural and industrial wastes to prepare light-weight aggregates is an effective way to maximize the use of natural resources and reduce energy consumption [42]. In general, these wastes have many characteristics, such as a large variety of chemical composition and significant land occupation. The rational and effective use of these resources can alleviate these inconveniences and reduce the consumption of non-renewable resources. Currently, the most frequently used waste to prepare lightweight to aggregate concrete is artificial aggregates, including oil palm shells, coconut shells, and drill cutting.

### 3.1. Agricultural Waste

Agricultural residues are extensively discarded or landfilled as solid wastes. Their utilization efficiency is quite low so that this waste accounts for a severe environmental problem. Recycling utilization of these wastes, such as oil palm shells, coconut shells, and corn cob, as lightweight aggregates to manufacture LWAC has brought broad interest in recent years.

**Figure 2:**
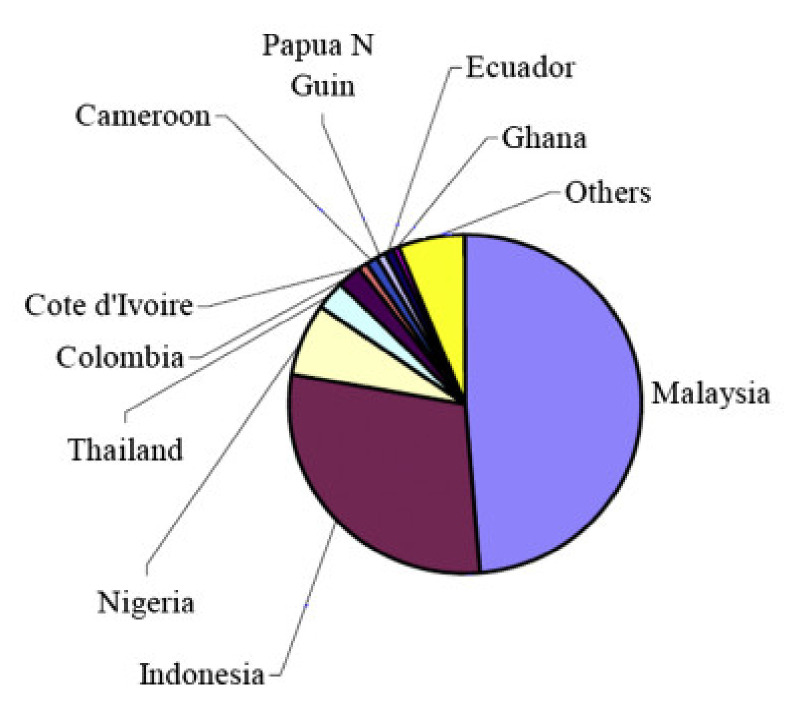
World palm oil production 1996–2000 [43], with copyright permission from Elsevier.

**Figure 3:**
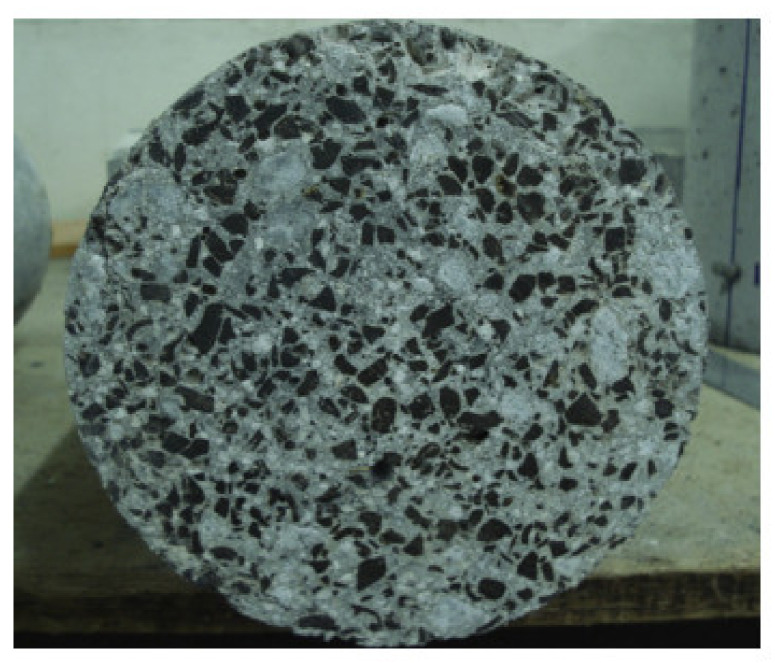
Oil palm shell (OPS) grains (darker) in lightweight concrete [49], with copyright permission from Elsevier.

Oil palm shell (OPS), as a kind of by-product of oil palm-based agriculture, has the potential to serve as the lightweight aggregate of concrete. The amount of waste OPS is huge, and the most majority of OPS is from many Asian countries such as Malaysia, Indonesia, and Thailand, as shown in Figure 2 [43]. Using OPS as LWA to fabricate concrete was first studied in 1985 by Abdullah in Malaysia [44]. Some researchers investigated the OPS concrete, and the results showed that, in most cases, the compressive strength of OPS (4.75–9.5 mm) lightweight concrete is higher than that of the structural lightweight concrete (20–35 MPa), and the density is about 20–25% lower than normal-weight concrete [45,46,47,48]. Recent studies have shown that the probability of producing high compressive strength OPS lightweight concrete is up to 53 and 56 MPa in 28 and 56 d respectively [49]. The fracture surface of a lightweight concrete specimen regarding OPS particles is shown in Figure 3.

Shafigh [50] used the oil-palm-boiler clinker (OPBC) lightweight fine aggregate (0–4.75 mm) instead of normal weight sand (by volume) to prepare the oil palm shell (OPS) lightweight concrete. The main properties, including workability, different types of density, compressive strength in different curing regimes, splitting tensile and flexural strengths, modulus of elasticity, water absorption and drying shrinkage strain of green lightweight concretes, were tested and discussed. They found that it is feasible to produce environmentally friendly lightweight structural concrete with high volume waste lightweight aggregate produced by the palm oil industry.

**Figure 4:**
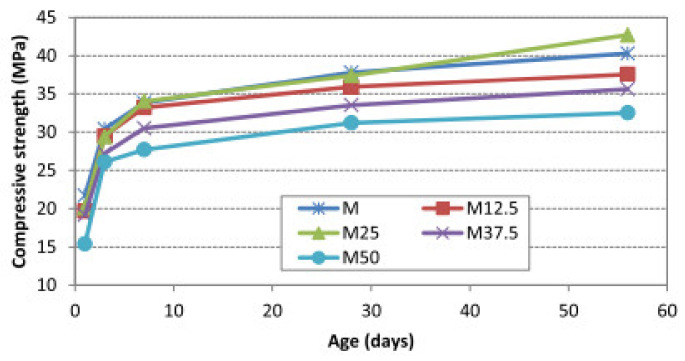
Development of the compressive strength of concrete [50], with copyright permission from Elsevier.

**Figure 5:**
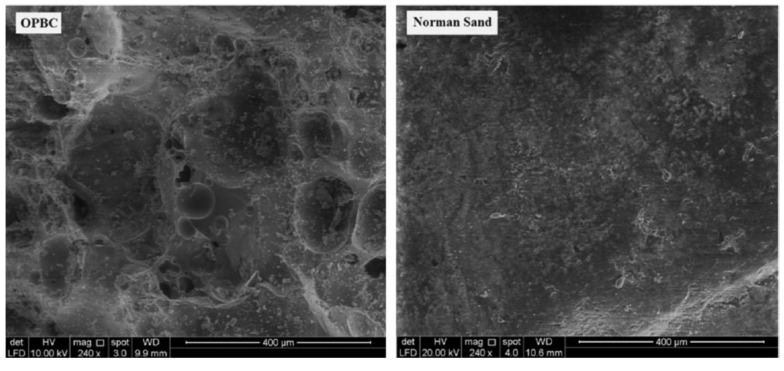
Scanning electron microscopy image for the surface texture of oil-palm-boiler clinker (OPBC) and normal sand [50], with copyright permission from Elsevier.

If the dry density was considered, the control OPS concrete was about 20.8% lighter than the normal-weight concrete. Using OPBC sand instead of ordinary sand will further reduce the density of the concrete samples. When the proportion of the OPBC sand were 12.5%, 25%, 37.5%, and 50%, the densities were 21.7%, 22.2%, 26.2%, and 27.4% lighter than ordinary concrete, respectively, shown in Table 1. Compared with ordinary concrete, OPS concrete with 0–50% OPBC sand has 21–27% lower density, and it should be noted that the OPS concrete with 37.5% OPBC sand (M37.5) reduced its compressive strength at each age by only 10–12%. The compressive strength development of all mixes up to 56 d is shown in Figure 4 [50]. Figure 5 is a scanning electron microscope image for the surface texture of OPBC and normal sand. It can be seen that the surface porosity of OPBC is larger than that of the ordinary sand, and a few holes can be observed on the surface. Therefore, when using the OPBC sand as aggregates, part of the water was absorbed by this lightweight sand, which increases the effective water-cement ratio, and thus leads to the reduction of the compressive strength.

**Figure 6:**
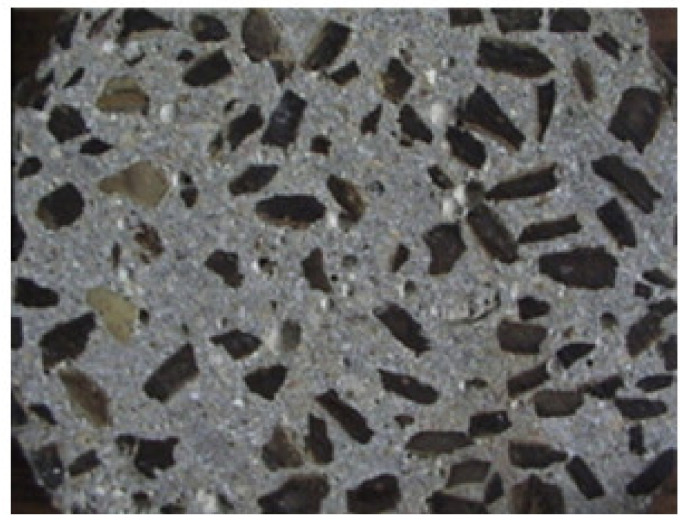
Cross-section of concrete made of coconut shells as coarse aggregate [53], with copyright permission from Elsevier.

**Figure 7:**
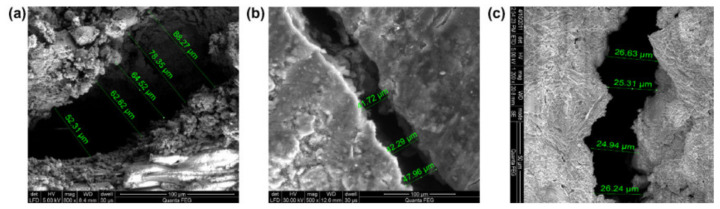
Crack width between cement paste and coconut shell aggregate (**a**) 3-day, (**b**) 7-day, and (**c**) 28-day [53], with copyright permission from Elsevier.

In addition to OPS, coconut shell (CS) is also a large amount of agricultural waste in the coconut industry. It can be used as coarse aggregates or as a potential building material in the construction industry, which is helpful to solve the environmental problem of using recycling solid waste [51]. It was found that the LWAC designed by waste also meets the essential strength required for use. In recent years, Gunasekaran et al. [52] explored various mechanical properties of concrete with coconut shells as coarse aggregate, including compressive strength, flexural crack tensile strength, and impact resistance. They concluded that the concrete using CS aggregate could satisfy the strength requirement of structural LWC. LWC developed from CS aggregate can be used for both structural and non-structural applications [52]. Gunasekaran demonstrated the ultimate bond strength of coconut shell aggregate concrete under all types of curing conditions was much higher than the theoretical bond strength [53]. Figure 6 shows the low magnification SEM crack images between the cement paste and the coconut shell aggregate. The bonding strengths between the cement paste and the coconut shell aggregate was studied. Figure 7 gives the microstructure of the cement paste and CS fractures cured for 3, 7, and 28 days, respectively. The width of the cracks between the CS and the cement paste shows a narrowing tendency from about 70 µm to 20 µm with increasing curing time.

Apart from OPS, corn cob, an agricultural solid waste from maize and corn was also widely incorporated with cementitious powder to prepare lightweight concrete. Previous studies [54,55] have shown that corn cob ash has over 65% SiO_2_ with the combination of Al_2_O_3_. This means that the corn cob ash can be used as a cementitious material. Corn cob as a lightweight aggregate for producing lightweight concrete in the non-structural applications was first researched by Pinto, and they measured the density, compressive strength, and thermal insulation properties of the corn cob LWAC. The results were compared with a non-structural lightweight concrete made with a lightweight artificial aggregate, namely, the expanded clay. The results pointed out that, although the compressive strength of the corn cob lightweight concrete is lower than the expanded clay lightweight concrete, the thermal conduction coefficient showed its advantages as non-structural applications [56].

The cork industry generates large quantities of waste, which has been studied for the application in the construction industry. In terms of grain density, the density and absorption of the loose body are comparable to that of expanded perlite, vermiculite, and other artificial light aggregates. Compared with expanded perlite or vermiculite, the use of cork particles as lightweight aggregate can reduce energy consumption, consume renewable resources, and be more cost-effective. Besides, cork has a high absorption value and can be used as a lightweight aggregate for internal curing of concrete. As for other physical properties, the advantages of cork as a lightweight aggregate are verified [57].

Besides these materials, Ozturk et al. [58] revealed the possibility of using tobacco waste to produce lightweight concrete tobacco ash as an aggregate, and Salas et al. [59] discussed the substitution of sand and gravel with rice husk in concrete.

### 3.2. Industrial Waste

Although the reuse of agricultural waste as an aggregate of lightweight concrete can achieve the acceptable performance of LWAC, its property still has room for improvement. Many industrial wastes include drill cutting, and steel cutting has been widely applied as aggregates.

Drill cuttings consist of a fine mix of rock particles produced by drilling for oil and gas during either exploration or production, and they will be contaminated with drilling fluids and contain high concentrations of chloride salts [60]. This limits the potential for using the original drill cutting samples in lightweight aggregate production as the formed products show high levels of leaching [61]. To reduce the leaching of chloride ions, Ayati et al. [61] washed the drill cutting, which can decrease the calcination temperature and improve the properties when it was used as aggregates in LWAC.

**Figure 8:**
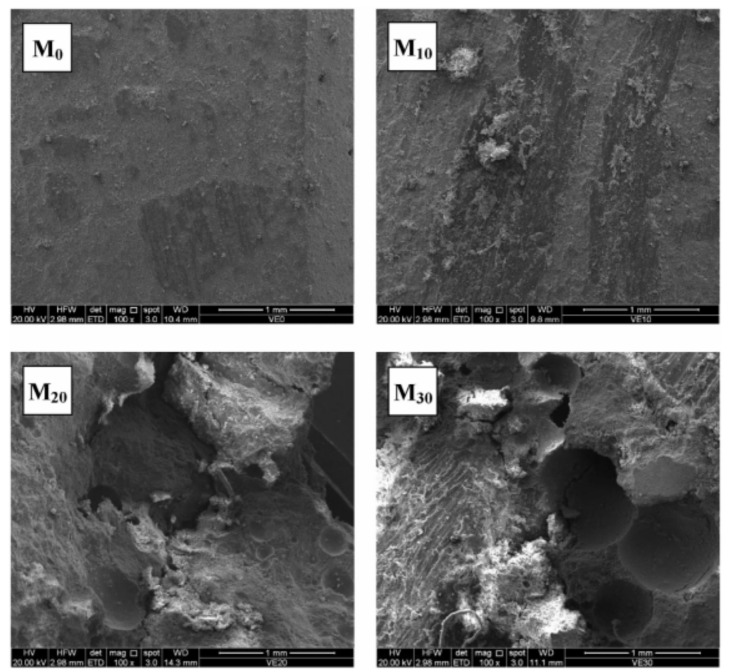
SEM images (100%) showing the morphologies of M_0_, M_10_, M_20_, and M_30_ [62], with copyright permission from Elsevier.

In addition to drilling cuttings, waste plastic is another recycled material as aggregates. Colangelo proposed polyolefin waste aggregate (PWA) refined from recycled plastic materials as LWA instead of natural aggregate in the manufacture of LWAC [62]. It can be predicted that the addition of PWA provides an opportunity for the production of LWAC to reduce the impact of plastic materials on the environment pollution, thereby promoting the development of ecologically sustainable buildings. Figure 8 illustrates the SEM images of the interface between the waste plastic aggregates and cement binder. It exhibits a satisfactory adhesion and good compatibility with the cement matrix.

Steel cutting is another under-utilized resource. El-Sayed [63] did research on the re-utilization of industrial lathe wastes. These lathe wastes were used to replace steel fibers because of their low price and convenient to obtain. Lo et al. [64] and Zhang et al. [65] used high-carbon fly ash and furnace bottom ash after sintering as LWA. Through the suitable manufacturing method to make LWA and proper proportions can let LWAC achieve equivalent workability and similar compressive strength to that of the NWC.

### 3.3. Challenges

It was claimed that if the raw materials were appropriately selected, the LWAC would show its distinctive advantage compared to the NWC [66]. However, the LWA selection criteria, the mixture designing policy, and the curing conditions have significantly influenced the strength and the durability of the LWAC. Although recent studies have shown that, with specific materials and appropriate designs, LWAC can satisfy the strength requirements and be used as structural applications, the conflict between the bulk density and the mechanical properties is still the main challenge of the LWAC structures. Meanwhile, the applications of LWAC as non-structural elements in constructions need to be further investigated. This is an effective route to expand the application of the LWAC.

To improve the mechanical properties of LWAC, reducing the water-binder ratio is a widely used method. However, when the coarse aggregate is replaced by the lightweight aggregate, the volume of the aggregates will be significantly increased, and the water-binder ratio will be considerably reduced. This brings a negative impact on the workability of the LWAC. Pierce et al. [67] used crumb rubber in flowable fill to produce an LWAC. The results showed that rubber powder can serve as a complete substitute for concrete sand and inflow filling without significant separation of rubber [68,69]. Thus, how to select an appropriate LWA, which can effectively mitigate the workability problem, is worthy of investigation.

Although many industrial and agricultural wastes have been recycled and reused for manufacturing lightweight aggregates, or as supplementary cementitious materials to replace Portland cement, their utilization efficiency is still quite low. As a result, how to high-effectively reuse these wastes remains a significant challenge. Furthermore, in the actual production process, using industrial waste to manufacture aggregate often needs additional processing, which inevitably consumes energy and causes secondary pollution. Exploring environmentally friendly and cost-effective approaches are imperative to develop green LWAC.

## 4. Mechanical Properties of Green LWAC

**Figure 9:**
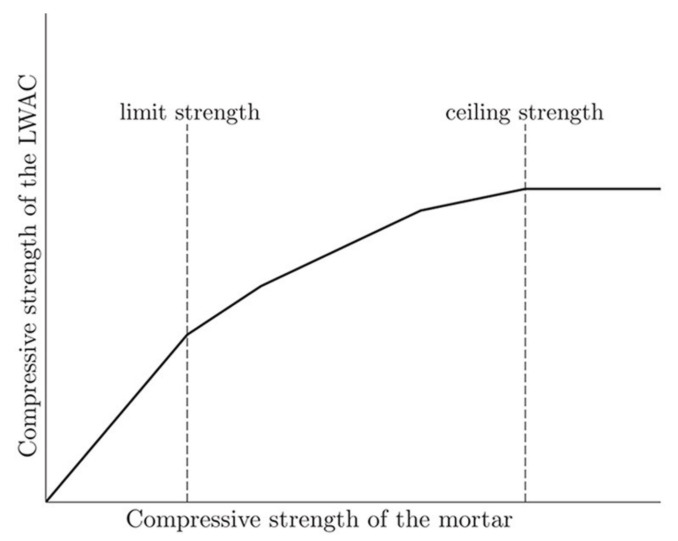
Relationship between the strength of the mortar and of light weight aggregate concrete (LWAC) made with the same mortar [70], with copyright permission from Elsevier.

Concrete composite can be considered as a two-phase material, i.e., the aggregates embedded in the cementitious binder phase. According to the properties of aggregate and mortar, concrete can be divided into two categories. In the first type of concrete, including NWAC and some LWAC, the relatively strong aggregates are embedded in the relatively weak cementitious binder matrix. The short-term strength mainly depends on the water-cement ratio and the cementitious binder. In the second type of concrete, the relatively weak aggregates are embedded in the relatively strong cementitious binder matrix; hence, the influence of the aggregate on the strength of the concrete must be considered. By comparing the relationship between the mortar strength and the concrete strength, the influence of the aggregate on the overall compressive strength of concrete can be evaluated, as shown in Figure 9. In this figure, the limit value represents the change from type one to type two. When the compressive strength is lower than this value, the LWAC shows the same behavior as ordinary concrete, however, when the compressive strength is higher than this value, the stress distribution in the LWAC varies because of the properties inversion of the two main components. The ceiling strength value represents the tensile strength of a particular aggregate whose weakness can be compensated by increasing the strength of the mortar [70].

Yap [71] aimed to compare the mechanical properties of the normal weight concrete and LWAC. By using oil palm shell (OPS) and broken granite aggregates as coarse aggregates, steel fibers as reinforcement, the LWAC were prepared with the mix proportions being listed in Table 2. The results showed that the compressive strength of the OPS concrete and NWC are comparable. The splitting tensile strength and the modulus of elasticity of the OPS concrete are slightly lower than the normal weight concrete. The addition of the steel fiber is beneficial to enhance the tensile strength and the brittleness. The mechanical properties, including the compressive strength, flexural strength, brittleness ratio, and elastic modulus of each test piece are shown in Table 3. In addition, the torsional ductility of the OPS concrete is over two times higher than the normal weight concrete.

The mechanical properties of the LWAC made of oil palm shell (OPS) as the lightweight aggregates had been tested by Teo [72]. The results demonstrated that the compressive strength, splitting tensile strength, modulus of rupture, and modulus of elasticity of the hardened OPC concrete have reached 28 MPa, 2 MPa, 5 GPa, and 5.3 MPa, respectively. These values were about 30% higher than the requirements of LWAC as structural lightweight concrete, and barely biological decay was observed after 6 months. In addition, the spitting tensile strength and the modulus of rupture is approximately 8% and 18% of the compressive strength, which are comparable to other lightweight aggregate concrete. The bonding strength testing results demonstrated that the experimental bonding strength of the OPS concrete is much higher than the theoretical bonding strength stipulated in BS8110. Another study demonstrated that that the 28 days compressive strength can reach a similar value as normal concrete by using OPS as lightweight aggregates and the 56 days compressive strength was even 6% higher than the control sample. Although the splitting tensile strength of the OPS concrete is about 20% lower than the normal weight concrete even with the same compressive strength, the testing results confirmed that all samples made of OPS as lightweight aggregates had the splitting tensile strength value higher than 2 MPa, which means all samples are qualified as the structural elements [50].

After the investigation of the OPS, another study claimed that using palm oil clinker (POC) to replace the OPS as coarse aggregates has a positive impact on the mechanical properties of the LWAC [73]. In this study, the OPS were replaced by POC with contents of 25%, 50%, 75%, and 100%, and the corresponding compressive strengths are 14.7%, 19.8%, 28.5%, and 43.1% higher than the control samples. In addition, the modulus of elasticity of the POC concrete is about 2.5 times higher than the control sample, because of the high bonding strength between the mortar and the aggregates. The highest modulus of elasticity reached about 35 GPa with 100% POC aggregate.

The application of granulated cork with bark (GCB) as lightweight aggregate to prepare concrete has been investigated most recently [57]. In this study, the chemical compositions and the physical properties of the GCB were tested followed by characterizing its alkali-silica reactivity. The microstructure of the GCB was also observed and analyzed via SEM/EDS and FT-IR. It was concluded that the mechanical properties of the GCB concrete are significantly determined by the curing conditions, surface morphology, particle shapes, and chemical compositions. This is a new agricultural waste that is worthy of further investigation as lightweight aggregates.

Apart from the agricultural wastes, many types of industrial wastes, including recycled concrete, recycled clay bricks, fly ash, and various types of sludge, were also used as lightweight aggregates to prepare LWAC. Bogas [74] investigated the mechanical properties of the LWAC by using recycling crushed lightweight structural and non-structural concrete, with densities below 2000 kg/m^3^, as aggregates. The mechanical testing results demonstrated that, with using the recycled concrete as lightweight aggregates, the compressive, splitting tensile strength, and the modulus of elasticity have increased 14%, 32%, and 22%, respectively. It was claimed that the recycled aggregates obtained from the non-structural lightweight concrete can satisfy the requirements and be used in structural LWAC.

The demolished waste clay bricks were used as lightweight aggregates to prepare LWAC [75]. The demolished waste clay bricks were crushed, cleaned, dried, and sieved before using as aggregates. With proper mixture design, the highest compressive strength can reach as high as 40 MPa, and the compressive strength increased with decreasing w/b ratio. The corresponding highest modulus of elasticity reached 25.6 GPa. It was found that the key factors that determined the mechanical properties are the w/b ratio and the volume ratio of fine aggregate to total aggregate. An interesting statement from this study is that, based on the microstructure analysis, it was found that the pozzolanic reaction between the waste clay bricks and the binder reinforced the interfacial zone. The rough surface and the pores optimized the microstructure, and reinforced the weak zone of the LWAC.

Except using the recycled construction materials as lightweight aggregates, using industrial wastes to prepare lightweight aggregates is another important branch of the LWAC manufacturing method. Lo [64] prepared lightweight aggregates by using the high-carbon fly ash and clay as raw materials. The loss of ignition was as high as 16.3%, and the contents of the SiO_2_, Al_2_O_3_, Fe_2_O_3_, CaO, MgO, K_2_O, and Na_2_O are about 46%, 18%, 9%, 5%, 1%, 2%, and 2%, respectively. The 28 days compressive strength and splitting tensile strength can reach 56 MPa and 3.0 MPa, respectively, and the measured modulus of elasticity is about 19 MPa. An interesting phenomenon being observed in this study was the 7 d compressive strength had reached 91% of the 28 d compressive strength. This was the reason that the loading transfer mechanism between the hydrated cement paste and the lightweight aggregates. The microstructure analysis of the ITZ revealed that the cement paste had penetrated into the lightweight aggregates, which reinforced the interfaces between the aggregates and the cement paste.

Sludge ceramsite (SC), recycled aggregates (RA), and ground granulated blast-furnace slag (GGBS) were used as coarse aggregates, fine aggregates, and supplementary cememtitious materials to prepare the LWAC [76]. The main chemical compositions of the sludge ceramsite are: SiO_2_ (55%), Fe_2_O_3_ (14.3%), Al_2_O_3_ (20.6%), K_2_O (2.7%), CaO (2.7%), MgO (1.4%), and balance (3.5%). This study investigated the synergistic impact of the aggregates and cememtitious binder. The sludge ceramsite was 100% used as the coarse aggregates and the cement was partially replaced by GGBS. It was found that, although using the SC along with RA and GGBS had significantly reduced the compressive strength, if solely using SC as coarse aggregate, the compressive strength of the LWAC can reach about 13.9 MPa. The elastic modulus results of all samples were in the range of 6.5–9.5 MPa. Unlike the statement from Zhao’s study [75], the microstructure analysis of this study demonstrated that the weak part of the LWAC was the sludge ceramsite rather than the ITZ area under compression. Appropriate amount of GGBS had a positive effect on the mechanical properties development of the LWAC because of the hydration of GGBS and the filling effect of the GGBS particles, but the dosage is quite critical. The highest amount GGBS was recommended as 30%.

**Figure 10:**
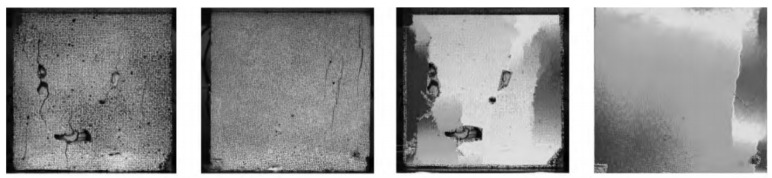
Typical failure modes of cubic specimens [77], with copyright permission from Elsevier.

From compression and crack development perspectives, microcracks often occurred at the interface between the binder and the aggregate. As the stress increases, the cracks in the vicinity of aggregates gradually expanded, and the cracks in the mortar interlocked each other until the failure occurred, as demonstrated in Figure 10 [77].

**Figure 11:**
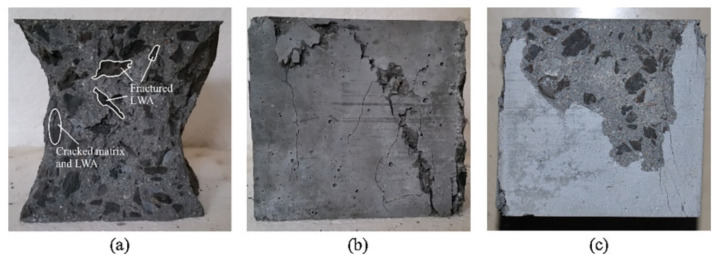
Compression failure mechanisms: (**a**) plain LWAC specimens, (**b**) steel fiber LWAC specimens, (**c**) carbon fiber LWAC specimens [82], with copyright permission from Elsevier.

It is not surprising that the mechanical properties of the LWAC is relatively lower than NWC, and the strengths of the LWAC decrease with increasing content of LWA [16,78,79,80,81]. As result, various types of fiber were used as reinforcement to improve the mechanical properties of lightweight concrete. Wu et al. [82] investigated the effect of fiber on fracture morphology under compressive loading. In this section, two types of steel fiber (SF) and carbon fiber (CF) were used to improve the mechanical properties of LWAC. The fracture morphology was characterized, and the failure mechanisms were analyzed, as shown in Figure 11. For plain LWAC, under compression loading, vertical cracks first appeared in the middle and high parts of the cube specimen, and then propagated with the increasing of compressive force [83]. Under the compression force, vertical cracks can be clearly observed on the surface of the plain concrete. Compared with the plain LWAC, the steel fiber-reinforced LWAC (SFLWAC) and carbon fiber enhanced LWAC (CFLWAC) had no significant flaking, which was attributed to the fiber bridge effect. Besides, after the fracturing of concrete, the surface of the specimen was maintained well, which means that there was no large amount of vertical fracture in the test process, and the addition of fiber can be used to restrain the lateral deformation [82,84]. It is noteworthy that the fiber specimens have irregular crack growth paths due to the fiber fracture or pulling out. This indicates that their energy dissipation capacity is positive to enhance the mechanical properties of LWAC [82,85].

## 5. Shrinkage

Shrinkage is one of the primary reasons that account for the cracks. The early age cracks of cement-based materials will lead to premature degradation of concrete structure, so as to shorten its service life. To reduce shrinkage and improve resistance to shrinkage cracking performance of cement-based materials, a variety of methods were adopted, including (1) expansive-based cement/admixture which compensate the shrinkage through the expansion-induced stress [86,87]; (2) internal curing effect originated from the lightweight sand (LWS) and lightweight aggregate, which resulted in water absorption, and mitigate the autogenous shrinkage [88,89]; (3) shrinkage reducing admixture (SRA) to reduce autogenous and drying shrinkage [90,91], (4) the reduction of the surface tension of pore solution is also a way to inhibit shrinkage and cracking; and (5) the addition of fiber can effectively improve the tensile strength and tensile creep behavior, so as to strengthen shrinkage cracking resistivity and decrease crack width [92,93]. Compared with NWC, the pre-wetting LWA makes concrete a lower early cracking sensitivity, which can significantly reduce the risk of premature cracking of concrete. So the moisture content of aggregate has a great influence on early cracking [94].

### 5.1. Internal Curing

Internal curing, also known as self-maintenance curing, refers to a curing method that relies on pre-absorbent materials to release water to maintain sufficient moisture inside the concrete under adiabatic conditions, it was developed to eliminate self-desiccation and reduce autogenous shrinkage [95]. In recent years, internal curing is considered to be a very effective method to increase the internal humidity of concrete and reduce self-shrinkage.

The self-shrinkage reduction mechanism of the lightweight aggregates is primarily from the gradually releasing of the internal water. During the hydration process, a humidity gradient is formed between the internal curing agent and the cementitious material, and the capillary pressure difference will be generated so that the moisture in the LWA will be released to compensate for the loss of humidity [96].

The effects of LWA performance and mixing parameters on the auto-shrinkage performance have been studied previously. Harmful effects of internal curing on the early strength had been reported in most cases [88]. However, the influence of internal curing on the strength of late age concrete is variable, which mainly depends on the type and content of the agent, the presence of chemical additives, and the content of aggregate. The results showed that LWA had a particularly adverse effect on the 28-day standard compressive strength and tensile strength, but the use of an appropriate amount of fine LWA had less impact [97,98]. Therefore, it is essential to find an optimal value between the reduced auto contraction and strength. The efficiency of internal curing aggregate depends on its pore structure and specific surface area. Equilibrium residual water is the effect of pore structure on aggregate efficiency. Meanwhile, the impact of surface area on water content can be reflected by the release rate of water [99].

Early age crack that emerged on cementitious materials caused by the autogenous shrinkage plays a significant role in determining the strength of concrete materials, especially for LWAC. Therefore, one of the best ways to reduce early age cracks in the cement matrix is the incorporation of SCMs. Meddah investigated the effect of SF on autogenous shrinkage of LWAC. The results showed that the greater the autogenous shrinkage, the greater the induced internal tensile stress, and the appropriate SCMs can increase the volume stability [100]. 

Bentz [89] established an equation to calculate the displacement level of saturated LWA, which can provide all the water needed for the complete curing of concrete. This calculation assumed no water exchange with the external environment (seal curing). Simulation results demonstrated that a good dispersion system with small saturated LWA particles is beneficial to the field concrete curing, which is similar to the gap protecting concrete from freeze-thaw damage.

### 5.2. Shrinkage Reducing Admixture

The shrinkage reducing agent is composed of polyether or polyalcohol organic matter or their derivatives [101]. The mechanism of the shrinkage reducing agent is to reduce the surface tension of water in the capillary pores to reduce the additional pressure in the process of water loss, thereby reducing the shrinkage of the capillary pores [102].

Shrinkage reducing admixture (SRA) is a water-soluble organic chemical with low viscosity, whose main function is to reduce the surface tension of the solvent [103]. At the beginning of the use of the SRA phase, it was considered that SRA will effectively reduce the surface tension, and then it can mitigate the drying shrinkage. Moreover, it was found that SRA successfully reduced the autogenous as well as drying shrinkage [104]. Sant et al. [105] proved that the SRA mixture led to the early expansion in the process of supersaturation and crystallization of calcium hydroxide amplification. Using SRA can reduce shrinkage and deformation, and thus improve the cracking resistance of the NWC. The relative reduction of internal cracks will help enhancing the durability of the concrete, and can increase the viscosity of pore water in NWC. However, it was barely used in LWAC and combined with the internal curing brought by the water content in LWA, which needs further investigation.

## 6. Durability

As for the research of lightweight aggregate concrete, most of them were focused on the aggregate types [106], mechanical properties [62,82] and interface transition zone [82], the durability of the LWAC manufactured with waste and recycled materials are still very limited.

### 6.1. Carbonation-Induced Corrosion

The carbonation of concrete is a diffusion process of carbon dioxide through the porous microstructure of concrete. The ingressed carbon dioxide reacts with the calcium hydroxide and forms calcium carbonate, which reduces the pH value of the pore solution as low as 11, and thus results in the corrosion of the steel reinforcement in concrete [107]. As well-known, the gas diffusion rate of LWAC is higher than that of the NWAC because of the large porosity of the LWA, which leads to the fact that carbonization rate of the LWAC is faster than the NWC. As a result, the corrosion resistance of LWAC is strongly dependent on the porosity of the aggregate and the quality of the cementitious binder phase.

**Figure 12:**
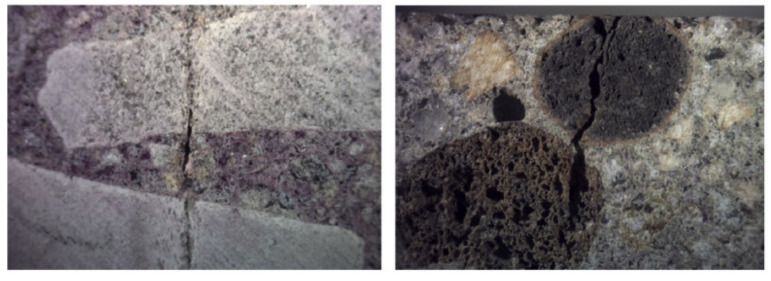
Natural crack crossing through the aggregate in NWC (**left**) and LWAC (**right**) [110], with copyright permission from Elsevier.

Although LWAC exhibits excellent performance, its carbonization mechanism is still lacking to the best of our knowledge. Newman [108] pointed out that LWAC’s carbonization resistance could be similar to the NWC if LWAs were properly protected by high-quality paste in concrete, and this conclusion was supported by Bogas [109]. It was pointed out, the carbonation resistance of LWAC reduced with the escalation of the aggregate’s porosity [110]. In this study, two types of lightweight aggregates, which were made of expanded clay and expanded slate, were used to assess the impacts of pores and cracks on the carbonization resistance of the LWAC. The effects of artificial cracks and natural cracks on the carbonation resistance of LWAC were compared with ordinary concrete with w/c ration of 0.55. It revealed that the artificial cracks have little effects on the carbonization process of this two LWAC. However, the natural cracks show a palpable impact on the carbonization rate of LWAC. The carbonization coefficient of LWAC with high porosity aggregates was 57% higher than that of the NWC (listed in Table 4). As shown in Figure 12, when the crack propagates, the crack width enlarged in the porous region of the aggregate; therefore, the crack width of the LWA is larger than the crack width of the encompassed mortar. This resulted in a severe carbonization behavior of LWAC. According to the relationship between the carbonation depth and the crack length, the carbonation rate with exposure to real condition is about 80% higher in cracked concrete. Although this study investigated the relationship between the carbonization and the porosity and cracks of the LWAC, the impacts of the aggregates chemical components on the carbonization resistance were not deeply discussed.

Zhao recycled the waste clay bricks as lightweight aggregates for the LWAC preparation [75]. The waste clay bricks were collected from a demolition site in Nanjing. After grinding and sieving, the waste clay bricks were used as the coarse and fine lightweight aggregates. The aggregates were all pre-wetted and for 24 h and surface dried before preparing the samples. The w/b ratio varied from 0.2 to 0.4. The carbonation process was implemented in a carbonation chamber at room temperature with 70% humidity and 20% of carbon dioxide concentration. The testing results demonstrated that the carbonation resistance of the LWAC decreased with increasing w/b ratio. Except the w/b ratio, the volume ratio of the fine aggregate (V_RS_) to the total aggregate is another factor that determined the carbonation resistance. It was claimed that the increase of the V_RS_ can slightly escalate the carbonation of the LWAC. The main contribution of this study is that the potential mechanisms of the impacts were pointed out. It was believed that the high w/b ratio will lead to the high porosity, which increase the potential carbonation degree of the LWAC, and the increase of V_RS_ will result in a high specific surface area of the waste clay brick, which enhance the pozzolanic reaction between the Ca(OH)_2_ and the clay bricks. This will dwindle the amount of the Ca(OH)_2_ in cementitious binder and thus mitigate the carbonation problem of the LWAC. This mechanism is worthy of further systematically investigation.

Apart from the w/b ratio, the mineral admixture is another important factor that determines the carbonation resistance of the LWAC. Gao [111] investigated the impacts of mineral admixtures on the carbonation resistance of the LWAC, and the results indicated that the carbonation resistance of the LWAC is significantly different with various mineral admixtures. In this study, grade II fly ash, pulverized fly ash, granulated blast furnace slag, and steel slag powders were selected as typical mineral admixtures to replace Portland cement and the shale haydite was used for lightweight aggregate. The type II fly ash and the steel slag powder had negative impact on the carbonization resistance. This originated from the internal pH value reduction of the pore solutions. Meanwhile, the addition of 20% of pulverized fly ash and granulated blast furnace slag showed a slightly negative impact on carbonation resistance of the 14 days samples, but a palpable positive impact of 28 days samples by comparing with the control samples. This phenomenon was derived from the pore filling effect that block the water and air penetration.

### 6.2. Permeability

**Figure 13:**
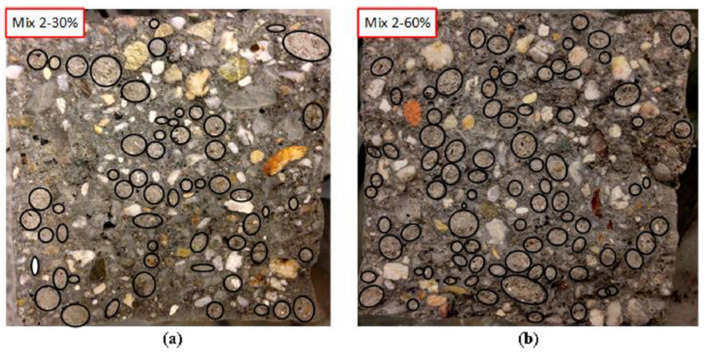
The cross-section of the concrete cube with (**a**) 30% and (**b**) 60% vol. aggregate replacement [114], with copyright permission from Elsevier.

In the physical and chemical deterioration mechanism of concrete, chloride ion and/or other corrosive ions diffusion in concrete is the most noteworthy phenomenon. The microstructure of concrete is the main factor controlling this process, which is related to water transport and ion diffusion in concrete [112]. The porosity of LWA determines its high water absorption rate [113]. Therefore, the water penetration of LWAC is generally higher than NWC, which is also proved in Tang’s study [114]. In this study, three types of artificial lightweight aggregates were manufactured by industrial solid wastes, including municipal solid waste incineration, bottom ash fines, paper sludge ash, coal fly ash, and washing aggregates sludge. It can be seen from Figure 13 that there are some pellets near the edge of the concrete sample, and the artificial lightweight aggregate produced by industrial waste is different from the natural aggregate in particle shape, so the distribution in concrete is not ideal. The water penetration depth in concrete with LWA consists of the penetration in the cementitious matrix in concrete, and the penetration in the LWA, while in normal concrete, it only had the former one. Therefore, the water penetration depth of the lightweight aggregate concrete is higher than the NWC. It was resulted from the concrete viscosity. It was recommended to decrease the w/c ratio or using chemical admixtures when using these lightweight aggregates.

Güneyisi et al. [115] used silica fume (SF) and metakaolin as mineral admixtures to enhance the permeability resistance of concretes. It was reported that the use of artificial lightweight aggregate causes an increase in gas permeability. To evaluate the corrosion behavior of LWAC, the accelerated corrosion test method was adopted by Güneyisi [116]. At the time of the sharp rise in the current, the concrete develops rapid longitudinal cracks. Adding SF can effectively delay the time of crack appearance as long as the appropriate dispersion was applied in the mixture [74,117].

### 6.3. Freeze-Thaw Resistance

**Figure 14:**
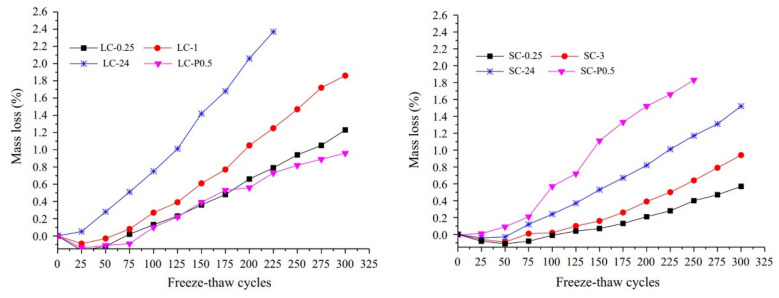
Effect of aggregate saturation degree on mass loss [118], with copyright from Elsevier.

**Figure 15:**
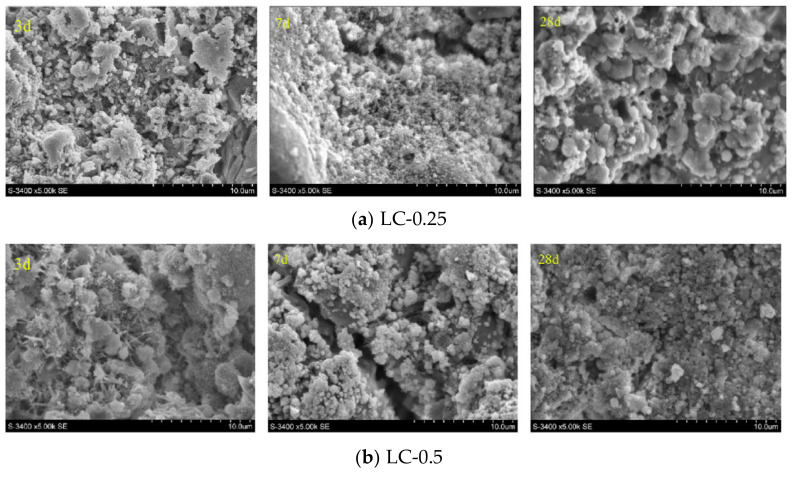

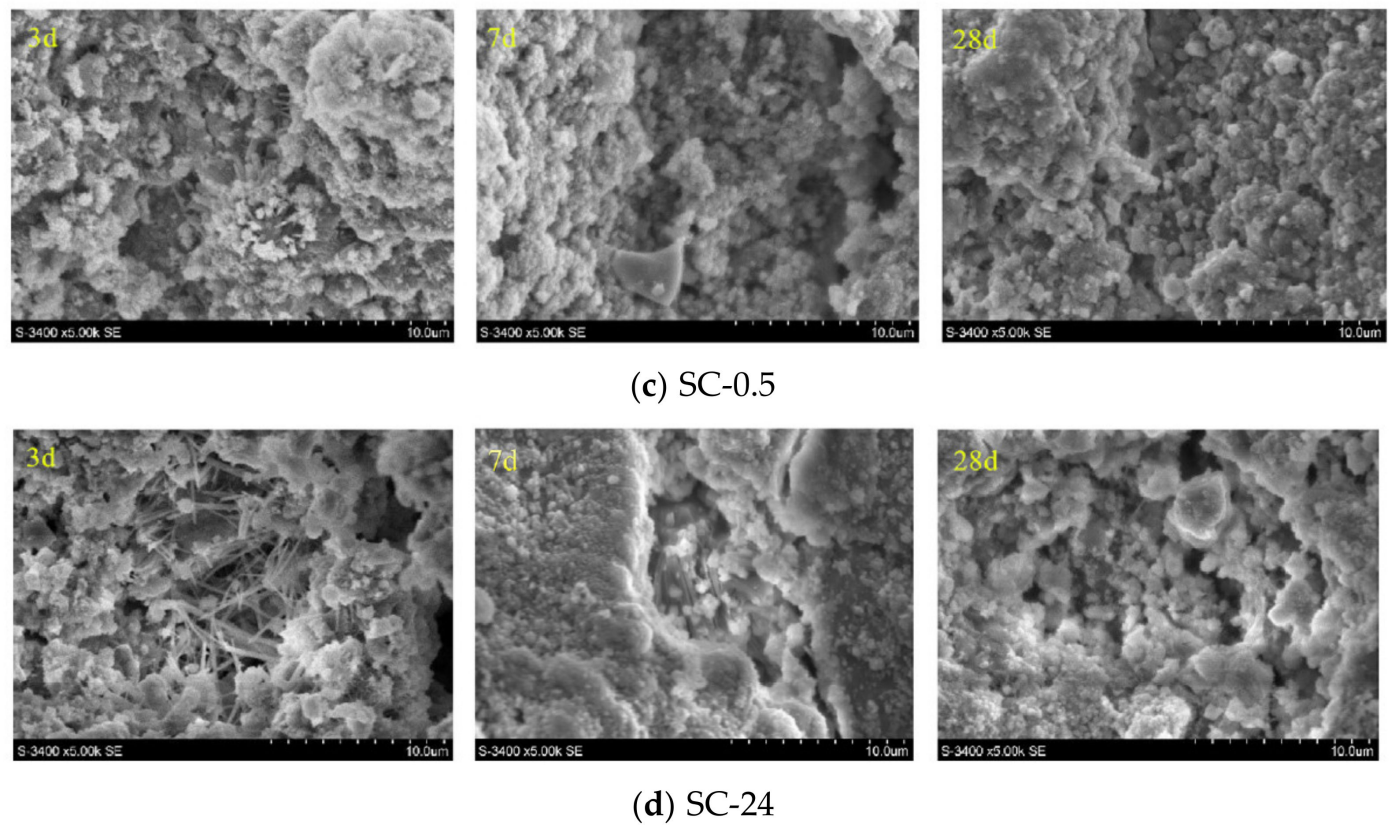
The SEM images of ITZ between the LWA and matrix: (**a**) Lytag LWA pre-wet for 0.25 h; (**b**) Lytag LWA pre-wet for 0.5 h; (**c**) Shale LWA pre-wet for 0.5 h; (**d**) Shale LWA pre-wet for 24 h [118], with copyright permission from Elsevier.

The water saturation condition of the LWA is the main factor affecting the freeze resistance of LWAC. Zhu [118] carried out a series of tests to investigate the impact of various pre-wetting time on the freezing-thawing performance of the LWAC. Lytag and Shale were selected as lightweight aggregates to prepare the LWAC. Figure 14 demonstrates the mass loss of the LWAC as a function of the freeze-thaw cycle. It can be seen from the figure that, the mass loss of the specimen increases with increasing the aggregate saturation degree, indicating that the high saturation has negative impact on the freeze-thaw resistance of LWAC. It can be seen from Figure 15 that when the curing time increased to 28 d, the microstructure of the hydration products have become very dense and the interface between the cement matrix and the aggregate is hard to be observed. In addition, little macroscopic crack and pores are quite small. It can be concluded that the change in aggregate saturation has no significant effect on the microstructure of the 28 d ITZ after the sufficient hydration reaction of the cement composite.

In Tang’s study [114], lightweight aggregates were manufactured from several industrial wastes, including municipal solid waste incineration, bottom ash fines, paper sludge ash, coal fly ash, and washing aggregates sludge. Before mixing with the cement, the lightweight aggregates were soaked in water for 24 h and air dried for 2 h to avoid the water absorption during mixing. After the freeze-thaw testing, the cumulative mass loss of the LWAC can be characterized in two types. One is the cumulative mass loss linearly increases with increasing freeze-thaw cycles, and another one is the cumulative mass loss had a two-stage variation, namely increased at the early cycles and kept stable in the late cycles. Unfortunately, it was claimed that the correlation between the freeze-thaw resistance and the properties of the aggregates is still unclear and needs to be further systematic investigation.

## 7. Advanced Characterization of LWAC

Acoustic emission (AE) is a relatively new method that can be used to explore the mechanism of explosive spalling of LWAC at high temperature [119,120]. The Gutenberg–Richter (GR) law is widely used to describe the amplitude distribution of AE signals [121].

(3) m=loga

(4) logN=a−bm

where *m* is the magnitude as defined in seismology; *m* is equivalent to the log scale of the amplitude “*a*” of the AE signal. *N* is the number of signals with a magnitude greater than *m*, and the coefficient b is the negative slope of the log*N*–*m* plot.

Specifically, the coefficient index *b* (the so-called *b* value) varies with the type of damage. At the initial stage, a large number of low-amplitude acoustic emission signals were generated. Later, fewer signals were produced, while the amplitude was high. This means that the value of *b* decreases gradually as the sample approaches the impending failure. This is at the heart of the so-called *b*-value analysis used to assess damage [120]. By monitoring the explosion spalling process, the steam pressure and temperature of different AE events are compared, which is helpful to clarify the mechanism of the explosion spalling process.

Means of neutron radiography (MNR) can be used for the visualization and quantification of liquid water transport [122]. The high sensitivity of neutrons to hydrogen atoms allows us to accurately measure changes in the moisture content of LWAC. The spatial and temporal variation of water content distribution shows that different water absorption directions and initial water content state have different impacts on the liquid water migration [123,124,125]. Wyrzykowski et al. [126] studied plastic shrinkage of mortars with SRA and LWA by such a method.

Non-destructive techniques (NDTs) play an essential role in the quality control of new buildings and the evaluation of the service condition of existing concrete structures [127,128]. The rebound hammer (RH) method and the ultrasonic pulse velocity (UPV) method were used widely to evaluate the compressive strength of the LWAC [129].

## 8. Concluding Remarks and Future Trends

The research work in recent years provides a framework for further research on the improvement of the mechanical properties of LWAC produced by waste and recycled by-products. The physical properties of LWAC, mixing ratio design method, freshness, and hardening properties of LWAC were discussed.

It was found that many solid waste materials, from both industrial and agricultural by-products, can be used as supplementary cementitious materials and lightweight aggregate materials to prepare green lightweight aggregate concrete. Several main industrial by-products, including silica fume, fly ash, lime powder, ground granulated blast slag, and various sludge, have been investigated as replacement of Portland cement. The results indicated that, provided with proper design and manufacturing process, these industrial by-products can be used as cementitious materials to prepare green lightweight aggregate concrete.

Apart from the supplementary cementitious materials, it was found that, many types of agricultural by-products, including oil palm shell, oil palm-boiler clinker, coconut shell, corn cob, cork, and tobacco wastes, and several industrial wastes, including drill cuttings, waste plastic, recycled clay bricks, various sludge, and steel cutting can be used as lightweight aggregates for manufacturing green lightweight concrete. The mechanical properties testing results shows that the lightweight aggregate concrete manufactured from the above mentioned agricultural and industrial by-products can meet the field application requirements. Although the durability testing of the lightweight aggregate concrete is quite limited, several studies have shown that the durability of the lightweight aggregate concrete is strictly correlated to the porosity and the saturation degree of the lightweight aggregates.

Although previous studies have demonstrated that the green lightweight aggregate concrete made of solid waste materials have a great potential to be widely used in practical applications, they are still facing several critical challenges need to be further investigated:

(1) The chemical reaction mechanisms between the recycled aggregates and cementitious binders should be investigated systematically. So far, the chemical bonding between the aggregates and the cementitious binders remains unclear. Further studies should focus on the chemical analysis of the aggregates, cementitious binder, and the interfacial area to elucidate and characterize the chemical reactions of the various recycled lightweight aggregates and the cementitious binders.
(2) For the lightweight aggregate concrete in the construction, prewetting of the lightweight aggregate is an appropriate way to reduce water absorption and improve the workability. The interface wettability between the lightweight aggregate and the cementitious pastes needs to be investigated. The impact of the saturation degree of various recycled aggregates on the workability, mechanical properties, and even durability should be further investigated.
(3) The porosity of the aggregates is another critical factor that determines the final properties of lightweight aggregate concrete. So far, the relationship between the porosity of the recycled lightweight aggregates and the final properties of the lightweight aggregate concrete is still lacking, especially when the chemical compositions of the lightweight aggregates were different.
(4) At present, researches on lightweight aggregate concrete mainly focus on high strength, which is an inevitable trend of future development. However, the durability challenge and the segregation resistance in engineering applications are worthy of this concern.

## Figures and Tables

**Table 1:** Density of concrete [50], with copyright permission from Elsevier.

Mix No.	Density (kg/m^3^)	Saturated at 28 Days	Oven Dry
	Demolded		
M	1908	1996	1900
M12.5	1976	1989	1880
M25	1966	1993	1868
M37.5	1917	1948	1771
M50	1881	1914	1743

**Table 2:** Mix proportion of normal weight concrete (NWC) and LWAC prepared with OPSC [71], with copyright permission from Elsevier.

Mix	Cement (kg/m^3^)	Coarse Aggregate		Mining Sand (kg/m^3^)	Water (kg/m^3^)	Silica Fume (kg/m^3^)	Superplasticizer (kg/m^3^)	Steel Fiber (v.%.)	Aspect Ratio of Steel Fiber
		Granite Aggregate (kg/m^3^)	OPS (kg/m^3^)						
NWC series									
NWC	320	810	0	1030	200	0	0	0	-
NWFRC-55	320	810	0	1030	200	0	0	0.50	55
NWFRC-65	320	810	0	1030	200	0	0	0.50	65
NWFRC-80	320	810	0	1030	200	0	0	0.50	80
OPSC series									
OPSC-0	530	0	320	970	170	53	0.35	0	-
OPSFRC-55	530	0	320	970	170	53	0.35	0.50	55
OPSFRC-65	530	0	320	970	170	53	0.35	0.50	65
OPSFRC-80	530	0	320	970	170	53	0.35	0.50	80

**Table 3:** Densities and mechanical properties [71], with copyright permission from Elsevier.

Mix	Density (kg/m^3^)	Compressive Strength (MPa)	Splitting Tensile Strength (MPa)	Flexural Strength (MPa)	Brittleness	Modulus of Elasticity (GPa)
NWC series						
NWC	2369	31.7	3.89	4.37	7.25	17.23
NWFRC-55	2492	35.1	3.98	5.42	6.48	19.59
NWFRC-65	2467	36.5	3.95	5.14	7.10	21.43
NWFRC-80	2488	36.8	4.11	5.57	6.61	20.71
OPSC series						
OPSC	1970	32.8	2.83	3.81	8.61	13.25
OPSFRC-55	2068	34.4	3.63	5.74	5.99	16.24
OPSFRC-65	2041	35.6	3.85	5.79	6.15	14.72
OPSFRC-80	2070	37.0	3.91	6.04	6.13	15.48

**Table 4:** Main results obtained in the accelerated carbonation test for uncracked reference concrete and concrete with artificial cracks [110], with copyright permission from Elsevier.

Type of Concrete	w/c	*w_c_* (mm)	Accelerated Carbonation					
			*x*_c_/*x*_c,*UR*_ (mm)	*x*_c,*C*_ (mm)	L*_C_* (mm)	K_c_/K*_c_,_UR_*	R^2^	K*_c,C_*
			28 d	28 d	28 d	mm/y^0.5^		mm/y^0.5^
NWC	0.55	0	5.4	-	-	16.5	0.97	-
		0.1	5.2	11.7	9.8	16.3	0.97	60.8
		0.2	5.5	13.5	10.9	16.8	0.97	87.0
		0.3	5.2	15.3	12.6	16.3	0.98	113.3
LWAC with Stalite	0.55	0	5.9	-	-	18.6	0.97	-
		0.1	5.3	12.0	9.9	18.2	0.98	64.2
		0.2	6.2	15.5	13.0	19.2	0.96	92.0
		0.3	6.1	18.3	16.1	18.8	0.96	115.7
LWAC with Leca	0.55	0	7.9	-	-	25.9	0.98	-
		0.1	6.8	12.9	11.0	24.9	0.97	56.6
		0.2	8.3	16.5	14.0	24.8	0.95	78.6
		0.3	8.2	17.1	13.0	25.9	0.97	108.0
